# Isolation and Annotation of Xitlalli, a Cluster EK1 Actinobacteriophage Isolated Using Microbacterium foliorum

**DOI:** 10.1128/mra.01270-22

**Published:** 2023-01-19

**Authors:** Younus A. Zuberi, Angelica Diaz-Sanchez, Jennifer Gutierrez, Simon J. Jakubowski, Sanghamitra Saha

**Affiliations:** a Department of Natural Sciences, University of Houston-Downtown, Houston, Texas, USA; DOE Joint Genome Institute

## Abstract

Xitlalli is an actinobacteriophage that was isolated from soil using Microbacterium foliorum. Based on gene content similarity to phages in the Actinobacteriophage Database, Xitlalli is assigned to cluster EK1. The genome is 53,929 bp long and contains 52 protein-coding genes, of which 26% could be assigned functions.

## ANNOUNCEMENT

Actinobacteriophages can be found in environments such as soil and water. The isolation and characterization of new actinobacteriophages expand not only our understanding of their genetic diversity and evolution but also our ability to deploy bacteriophages as therapeutic agents for drug-resistant actinobacterial infections (1). Here, we report on Xitlalli, an actinobacteriophage that was isolated from a soil sample collected in August 2019 near the Buffalo Bayou in Houston, Texas (29.7675N, 95.3594W), using Microbacterium foliorum NRRL B-24224 and standard procedures (2). Briefly, the soil sample was suspended in peptone-yeast extract-calcium (PYCa) liquid medium, inoculated with Microbacterium foliorum NRRL B-24224, and incubated for 3 days at 30°C with shaking. An aliquot of the culture was filtered through a 0.22-μm filter, and the filtrate was plated on PYCa top agar containing Microbacterium foliorum and incubated at 30°C to yield Xitlalli. Xitlalli, which forms clear plaques, was purified through three rounds of plating.

Xitlalli DNA was isolated using the Promega Wizard DNA cleanup system. Sequencing libraries were prepared using NEB Ultra II Library kits and run on an Illumina MiSeq system (v3 reagents) to yield ~61,268 single-end 150-base reads, with 160× coverage. Raw reads were assembled and checked for completeness using Newbler v2.9 and Consed v23, respectively (3). Genome ends were determined by comparison with similar phages with known ends and verified by read start buildup using Consed v23. The genome of Xitlalli is 53,929 bp long and circularly permuted, with a GC content of 60.2%. Based on gene content similarity (GCS) of ≥35% to phages in the Actinobacteriophage Database (phagesDB) (https://phagesdb.org), as determined using the GCS tool (https://phagesdb.org/genecontent/) at the phagesDB, Xitlalli is assigned to phage cluster EK1, which has 18 phage members (4, 5).

Genome annotation was completed using DNA Master 5.23.2 (6), GLIMMER 3.02 (7), GeneMark 2.5p (8), Starterator (2), Phamerator (9), BLASTp (10), HHpred V3.2.0 (11), and the Phage Evidence Collection and Annotation Network (PECAAN) (https://blog.kbrinsgd.org/overview/). The genome contains 52 genes, 29 of which are found on the left one-third of the genome and are transcribed leftward. The remaining genes are located on the right two-thirds of the genome and are transcribed rightward. A total of 14 genes could be assigned putative functions. DNA metabolism functions are encoded by leftward-transcribed genes and include DNA primase/polymerase (gp2), RuvC-like resolvase (gp8), DNA polymerase I (gp10), and Cas4 family exonuclease (gp12). Several structure, assembly, and lysis functions are encoded by rightward-transcribed genes; most notable here is gene 30, which is 13,479 bp long. A homolog of this gene is conserved throughout cluster EK1, with a currently unknown function. No immunity repressor or integrase functions could be identified, suggesting that Xitlalli is unlikely to establish lysogeny. The orientation of the genes in Xitlalli is shared with other members of cluster EK1; genes on the left arm of the genome (genes 1 to 29) are transcribed leftward, whereas genes on the right arm (genes 30 to 52) are transcribed rightward (12).

### Data availability.

The complete genome sequence is available in GenBank under accession number MW055914.1, and the raw sequencing reads are available in the NCBI SRA under accession number SRX11207665.
